# A bibliometric review of positive psychology and well-being research in Africa

**DOI:** 10.3389/fpsyg.2024.1384362

**Published:** 2024-06-21

**Authors:** Angelina Wilson Fadiji, Itumeleng P. Khumalo, Marié Philipina Wissing, Richard Appiah

**Affiliations:** ^1^Department of Psychology, De Montfort University, Leicester, United Kingdom; ^2^Africa Unit for Transdisciplinary Health Research, North West University, Potchefstroom, South Africa; ^3^Department of Psychology, University of Johannesburg, Johannesburg, South Africa; ^4^Department of Psychology, Northumbria University, Newcastle upon Tyne, United Kingdom; ^5^College of Health Sciences, University of Ghana, Accra, Ghana

**Keywords:** bibliometric review, positive psychology, well-being, research trends, Africa

## Abstract

Positive Psychology rapidly developed into an influential field of study and intervention, initially situated in Psychology, and later becoming multidisciplinary. Research interest in the study of (psychological) well-being has gained global popularity, with increasing salience in Africa. Although the global trends of these developments are relatively well-known, a bibliometric analysis of positive psychology research in Africa was necessary to shed light on the present hotspots and trends and future trajectories in this region of the world. The data source of the present bibliometric analysis study was Scopus, from which Positive Psychology and well-being research literature from Africa between 1983 and 2023 were searched. Using biblioshiny and VOSviewer, the 622 extracted articles were analysed, from which findings about the current condition, research hotspots, and thematic developmental patterns could be made. Africa experienced an initial slow growth period from 1983 until 2005, after which a rapid growth in research productivity, relevance and impact was experienced. In this regard, the results show that the focal point of scientific productivity is South Africa, with the dominance of South African institutions, particularly the North-West University, from where most positive psychology research is produced and cited. Even with potential access to international journal, African researchers seem to prefer to place their publications in the regional journals such as Journal of Psychology in Africa and South African Journal of Psychology. The research reviewed tends to be characterised by more dominant thematic clusters of positive psychology, psychological well-being, and subjective well-being, with a focus on human individuals. An increasing concern for contextual factors and potential antecedents and dynamics of well-being is also observed. The findings provide a good map from which identification of future research priorities can be deduced. As such, we speculate that future positive psychology research in Africa ought to be concerned with the following: greater distribution and intercountry collaborations across the continent, questions of conceptual clarity of terms, better understanding of contextual factors which influence well-being, and well-being research embracing the complexity of bio-psycho-social-ecological well-being, and science concerned with health-promotion interventions.

## Introduction

The recent exponential growth of the study of (psychological) well-being, also described as Positive Psychology (PP), was arguably catalysed by the 1998 presidential address by Professor Martin Seligman marking his election as president of the American Psychological Association. As Donaldson (2011, p. 4) puts it, “the positive psychology theme of the 1998 American Psychological Association Convention was a huge success.” In Africa, the first and second positive psychology conferences took place at the North-West University, Potchefstroom, in 2006 and 2018, respectively. These significant events were accompanied by a proliferation of research, including the validation of positive psychological (PP) constructs (Keyes et al., 2008; Schutte et al., 2013; Appiah et al., 2020a; Khumalo et al., 2022a; Cromhout et al., 2023), evaluation of positive psychological interventions (Guse and Fourie, 2013; van Schalkwyk and Wissing, 2013; Appiah et al., 2020b) cross-cultural or contextual exploration of well-being constructs (Agbo and Ome, 2017; Dzokoto et al., 2019; Wissing et al., 2020, 2021; Schutte et al., 2022), synthesis and critical reviews (Mahali et al., 2018; Wilson Fadiji and Wissing, 2022) and theoretical investigation into what well-being entails (Wissing et al., 2019). The 2018 conference gave birth to the edited volume, *Embracing Well-Being in Diverse African Contexts: Research Perspectives* (Schutte et al., 2022), published by Springer.

In line with broader trends in psychological science, a bibliometric analysis of positive psychology publications from the inception of the field in 1998 to 2010, reported that 74.5% of the authors were affiliated with institutions in North America, 17.6% in Europe, 3.2% in Asia (mostly China), 1.4% in Africa (mostly South Africa), and 0.9% in South and Central America (Hendriks et al., 2019). Thus, approximately 94.5% of the research stems from Western countries, and only 5.5% from non-Western countries (Schui and Krampen, 2010). A more recent systematic review of 863 empirical articles about positive psychology studies published between 1998 and 2014, reported that 41% of the studies were conducted in the U.S., 24% in Europe, 7% in Canada, 6% in Australia. Implying that about 78% of the articles were conducted in Western countries, while indicating a trend towards greater global representation of research in positive psychology (Kim et al., 2018).

In a recent publication, Wilson Fadiji et al. (2022) argued for the necessity for an African-centred PP, whose knowledge contribution is embedded in an African socio-cultural context and is shaped through using qualitative research methods and bottom-up approaches of knowledge generation, and accompanied by a synthesis of existing literature. Regarding the latter, a substantive review of existing positive psychology research in South Africa was published in 2007 (Coetzee and Viviers, 2007). This effort was followed by Van Zyl and Rothmann’s (2014) literature review on happiness and happiness interventions, and later a scoping review of positive psychological interventions in Africa by Guse (2022). Wilson Fadiji and Wissing (2022) provided an overview of ongoing positive psychology research in Africa but did not utilize a citation analytic tool. This is akin to the work of Møller and Roberts (2021) on well-being and quality of life in Africa. Furthermore, Van Zyl et al. (2023) reported on a systematic review of critiques and criticisms of positive psychology, indicating the need for further conceptualisation and theorizing.

With respect to recent bibliometric reviews, Wang et al. (2023) undertook and reported a bibliometric analysis of positive psychology literature in the world from year 1999 to 2022. However, because their analysis relied solely on the terminology of positive psychology and had an international focus, it may have missed studies within the African context in which specific ‘Positive Psychology’ terminology was not used. As argued by Wilson Fadiji and Wissing (2022), many studies on PP within the African context frame their research more broadly as ‘well-being’, without necessarily using the ‘PP’ label. Even in this context, the scholarship of well-being studies is known to have predated the positive psychology movement launched by Seligman and Csikszentmihalyi (2000). To shed light on current developments and trajectory of PP and well-being research in Africa, in the present study, we sought to conduct a bibliometric review of PP and well-being research carried out in this region of the world. We recognise that well-being research predates present PP movement, and thus did not impose a date restriction to our search.

### The foci of some constructs and empirical studies in the growth of PP

The positive psychology movement, for which there are signs of it being beyond its third wave (Wissing, 2022), was ushered in by a predominantly positive-focus first wave (see Lomas et al., 2021). In this first wave, positive psychology was described as being about subjective experiences, positive individual traits, and civic virtues and institutions fostering better citizenship (Seligman and Csikszentmihalyi, 2000). While this aligns with the initial aims of the field, it had some problems. Diener’s (2009) observation was that PP then tended to ignore the negative in life and had an exclusive focus on the individual. Further, positive qualities, such as optimism and forgiveness, can sometimes be detrimental to well-being and requiring appropriate context (McNulty, 2011). Similarly, certain seemingly negative presentations, such as anxiety, may at times be beneficial to an individual’s well-being (Lomas and Ivtzan, 2016). The second wave of positive psychology responded to these and other limitations of the first wave. Among others, the field’s second wave acknowledged and interrogated the philosophical and conceptual complexities of the very idea of the initially claimed ‘positive’. Thus, this wave examined ways in which the field developed a more subtle understanding of the dialectical nature of flourishing, involving a complex and dynamic interplay of the positive and negative experiences (Lomas and Ivtzan, 2016). The developments in the third wave (Lomas et al., 2021) indicate a shift beyond the individual person as the primary focus and locus of enquiry. Accordingly, the field is presently concerned with a deep and critical view at groups, organisations, and broader systems, and explores various socio-cultural factors and processes which impact people’s well-being, from politics to economics. Beyond the third wave, Wissing (2022) envisages a new post-disciplinary research domain of “inter- or intradisciplinary well-being studies in its own right” (p. 1) which is not disciplinary bound to Psychology.

In line with these waves, some constructs have also been at the fore of PP research. For instance, in the study of emotion, multiple lines of progress are evident. The broaden-and-build theory has continued to evolve, revealing the short- and long-term benefits of positive emotions across various domains. These benefits encompass thoughts, actions, stress management, health outcomes, as well as physiological and neurological connections (Tugade et al., 2021). Positive affect has been associated with increased longevity, reduced disease incidence, enhanced recovery from illness, and overall better health (Pressman et al., 2019). Relatedly, happiness studies have examined strategies (experiments and activities) to improve happiness (Sheldon and Lyubomirsky, 2021). Veenhoven (2020) reviewed differences in happiness across nations and linked them to important questions about what governments can or cannot do to raise levels of happiness, thus reaching toward issues of public policy.

Shifting to life evaluation, how the future is construed was covered with work on optimism showing that those who expect good things to occur have higher well-being, better health, and higher quality social ties, partly attributable to how they cope with adversity (Mens et al., 2021). Detrimental consequences of hope were considered, while calling for greater work on the origins of hope and cultural issues. A noteworthy output is an edited collection on hope across cultures that brings together research on theoretical foundations, hope and mental health, hope and flourishing as well as some predictors of hope (Krafft et al., 2023). Similarly, a compilation of both empirical and theoretical contributions on quality of life in African societies was edited by Eloff (2019).

In Africa, contemporary research has focused on various eudaimonic concepts. For instance, studies on meaning in life have been conducted among students (Mason, 2013; Nell, 2014; Wissing et al., 2014; Khumalo et al., 2022b), urban adults (Wissing et al., 2020, 2021), rural communities (Wissing et al., 2019; Koen et al., 2022) and cross-cultural samples (Wissing et al., 2020). Additionally, there is research on relational well-being (Wilson et al., 2019; Cromhout et al., 2022), patterns and levels of flourishing (Keyes et al., 2008; Appiah et al., 2020a), religion and well-being (Selvam, 2013; Wilson et al., 2022; Khumalo et al., 2023) and harmony (Schutte et al., 2022). A notable body of research on hedonic well-being includes the exploration of children’s subjective well-being in South Africa by Savahl et al. (2017), happiness in Nigeria (Agbo and Ome, 2017), and life satisfaction among large South African cohorts (Møller and Roberts, 2021). A number of studies have also focused on assessment including a review on the translation of well-being instruments in Nigeria (Chukwuorji and Osondu, 2023) and validation in Egypt (Salama-Younes, 2017). Despite previous synthesis, to the best of our knowledge, no studies have attempted to analyse the trends in well-being and PP research in Africa using bibliometric analytic tools. Hence the present study aims to address this gap.

### Aim of the study

This study sets out to conduct a bibliometric analysis of PP research and related well-being constructs in Africa to better understand the field’s current condition, hotspots of research, and thematic developmental patterns.

## Methodology

The study’s data source was SCOPUS, which is one of the world’s most reliable citation database and contains numerous high-quality papers (Zhu and Liu, 2020). We conducted the search between the months of March and May 2023.

The foundational elements of PP were initially outlined by Seligman in 1998 (Seligman and Csikszentmihalyi, 2000; Seligman, 2011). Furthermore, the APA Thesaurus included the index phrase “positive psychology” (Tuleya, 2007) in June 2003. Topics within PP are often represented by these phrases such as “well-being,” “life satisfaction,” “positive emotions,” “happiness,” “quality of life” and so on. Despite the challenge to determine the eligibility of literature due to the abundance of terminology related to PP (Wang et al., 2023), we decided to use terms which broadly cover the scope of well-being research in Africa. They are provided in the next section.

### Data search and inclusion criteria

The research data for this study was collected and recorded on May 19, 2023, at approximately 4:30 pm, covering the period from 1983 to 2023. The search was conducted in the title, abstract, and keyword fields of the Scopus database. The following search string was used: “OR” and “AND” operators was: (TITLE-ABS-KEY ((“flourishing” OR “positive education” OR “meaning in life” OR “positive psychology” OR “positive functioning” OR “positive emotions” OR “emotional well-being” OR “emotional wellbeing” OR “positive and negative affect” OR “subjective well-being” OR “subjective wellbeing” OR “life satisfaction” OR “psychological well-being” OR “psychological wellbeing” OR “relational well-being” OR “relational wellbeing” OR “hedonic well-being” OR “hedonic wellbeing” OR “eudaimonic wellbeing” OR “eudaimonic well-being”)) AND TITLE-ABS-KEY ((“africa” OR “african”))). The result was 2,885 documents.

We then employed an advanced search process involving the following search string (TITLE-ABS-KEY ((“positive psychology” OR “positive functioning” OR “positive emotions” OR “emotional well-being” OR “positive and negative affect” OR “subjective well-being” OR “life satisfaction” OR “psychological well-being” OR “relational well-being” OR “hedonic well-being” OR “eudaimonic well-being”) AND (“africa” OR “african”)) AND (EXCLUDE (SUBJAREA, “medi”) OR EXCLUDE (SUBJAREA, “nurs”) OR EXCLUDE (SUBJAREA, “busi”) OR EXCLUDE (SUBJAREA, “bioc”) OR EXCLUDE (SUBJAREA, “envi”) OR EXCLUDE (SUBJAREA, “econ”) OR EXCLUDE (SUBJAREA, “neur”))) and 1,015 articles remained from the initial pool of documents.

Additional exclusion criteria were added as follows: AND (EXCLUDE (DOCTYPE, “bk”) OR EXCLUDE (DOCTYPE, “no”) OR EXCLUDE (DOCTYPE, “ed”) OR EXCLUDE (DOCTYPE, “le”) OR EXCLUDE (DOCTYPE, “er”)) AND (EXCLUDE (SUBJAREA, “MEDI”) OR EXCLUDE (SUBJAREA, “NURS”) OR EXCLUDE (SUBJAREA, “BIOC”) OR EXCLUDE (SUBJAREA, “AGRI”) OR EXCLUDE (SUBJAREA, “EART”) OR EXCLUDE (SUBJAREA, “IMMU”) OR EXCLUDE (SUBJAREA, “ENGI”) OR EXCLUDE (SUBJAREA, “COMP”) OR EXCLUDE (SUBJAREA, “PHAR”) OR EXCLUDE (SUBJAREA, “ENER”) OR EXCLUDE (SUBJAREA, “DECI”) OR EXCLUDE (SUBJAREA, “MATH”) OR EXCLUDE (SUBJAREA, “VETE”) OR EXCLUDE (SUBJAREA, “MATE”) OR EXCLUDE (SUBJAREA, “CENG”) OR EXCLUDE (SUBJAREA, “PHYS”) OR EXCLUDE (SUBJAREA, “CHEM”)) AND (LIMIT-TO (AFFILCOUNTRY, “South Africa”) OR LIMIT-TO (AFFILCOUNTRY, “Ghana”) OR LIMIT-TO (AFFILCOUNTRY, “Nigeria”) OR LIMIT-TO (AFFILCOUNTRY, “Kenya”) OR LIMIT-TO (AFFILCOUNTRY, “Uganda”) OR LIMIT-TO (AFFILCOUNTRY, “Botswana”) OR LIMIT-TO (AFFILCOUNTRY, “Algeria”) OR LIMIT-TO (AFFILCOUNTRY, “Ethiopia”) OR LIMIT-TO (AFFILCOUNTRY, “Zambia”) OR LIMIT-TO (AFFILCOUNTRY, “Tanzania”) OR LIMIT-TO (AFFILCOUNTRY, “Cameroon”) OR LIMIT-TO (AFFILCOUNTRY, “Egypt”) OR LIMIT-TO (AFFILCOUNTRY, “Mauritius”) OR LIMIT-TO (AFFILCOUNTRY, “Benin”) OR LIMIT-TO (AFFILCOUNTRY, “Gambia”) OR LIMIT-TO (AFFILCOUNTRY, “Lesotho”) OR LIMIT-TO (AFFILCOUNTRY, “Liberia”)). Following this, the titles and abstracts of the remaining articles were screened to determine if they do indeed explore positive psychological constructs. At the end of this stage of the analysis, 622 publications were retrieved and analysed using biblioshiny and VOSViewer. The study exclusively considered the English-language literature, specifically focusing on articles, book chapters and reviews. The article selection process was methodologically conducted to ensure alignment with our research scope. We applied manual exclusion to refine the selection, resulting in a precise and focused representation of the literature closely attuned to the search string used.

### Analysis methods

The raw data from the 622 documents identified in the Scopus search were saved in CSV and BibTeX formats. We used the bibliometric tools VosViewer and “Bibliometrix” package software for the data evaluation (Aria and Cuccurullo, 2017; Sganzerla et al., 2021). Bibliometrix was employed to display productivity, scientific trends, the most productive authors, and widely cited articles from the collected documents. For further conceptual analysis, the authors conducted a keyword co-occurrence network analysis. Based on the primary keywords, authors, and their connections, the VosViewer programme generated maps illustrating dominant keywords and thematic evolution in the field in Africa.

## Results of descriptive statistics

### Publication by year

**Figure 1:**
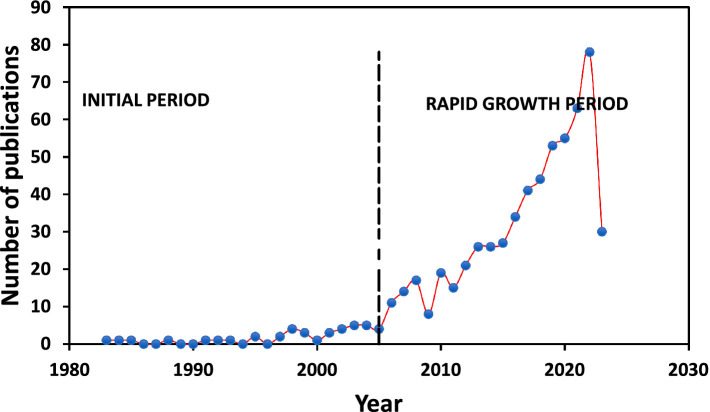
Publication by year.

**Figure 2:**
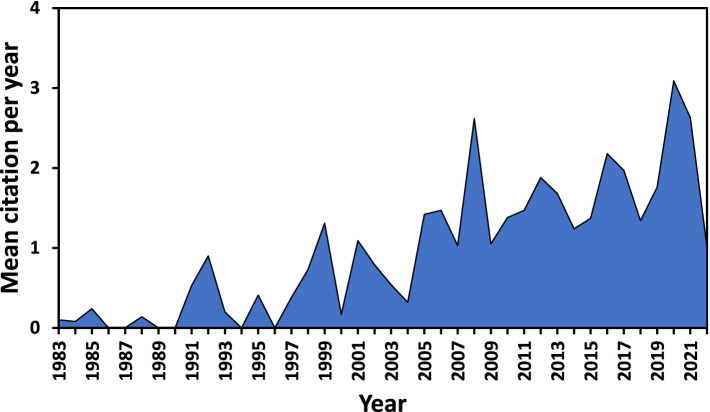
Average annual citations.

Figure 1 illustrates the annual distribution of the total of 622 scientific documents, depicting a growing trend since 1983, with a yearly growth rate of 8.87%. The figure is divided into two periods: the initial period and the rapid-growth period. In the initial period (1983–2005), the literature appears limited, with the maximum annual publication not exceeding 20 documents. The rapid-growth period (2005–2023) showed an exponential increase in yearly publication on PP and (psychological) well-being research in Africa, with almost 80 documents retrieved for 2023. Approximately 48 and 38% of articles published in 2021 and 2022, respectively, indicating a rapid and significant growth in the subject area over the period. Figure 2 illustrates the annual average of document citations, revealing that 2008 had the highest average number of document citations, followed by 2022. This finding aligns with the observed trends starting from 2005, which marked the beginning of the rapid-growth era, as depicted by the publications by year in Figure 1.

### Publication by country

**Figure 3:**
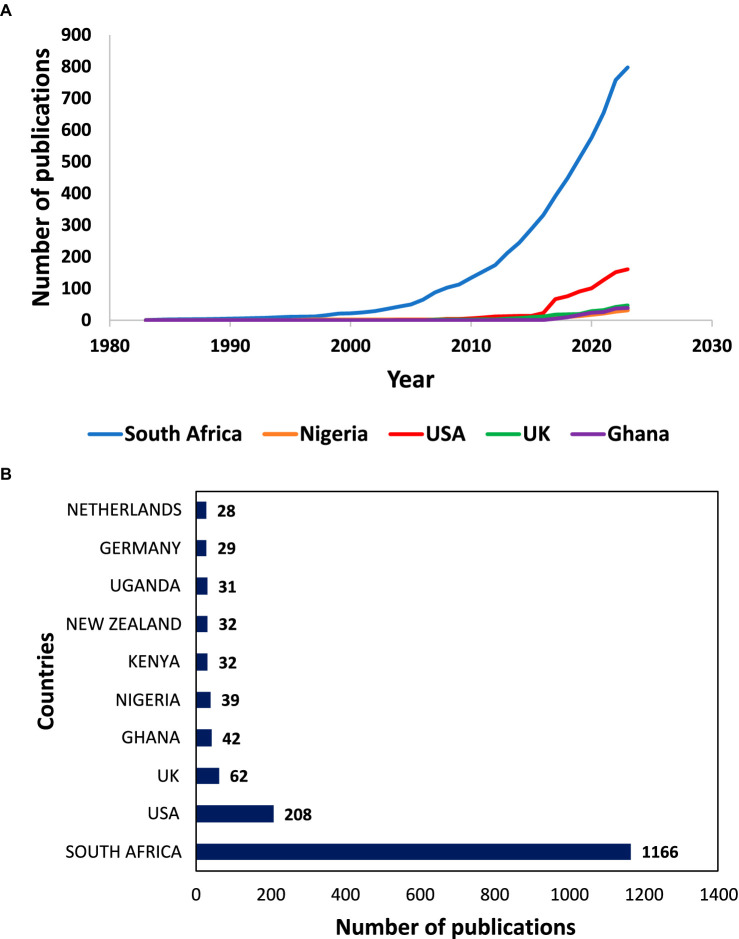
**(A)** Scientific publication by countries over time. **(B)** Number of publications by countries.

**Figure 4:**
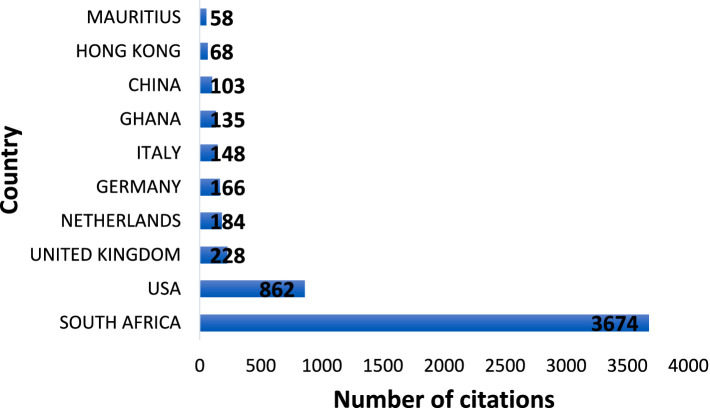
Number of citations by country.

Five countries were represented in the data on positive psychology and well-being in Africa. Figures 3, 4 illustrate the distribution of publication according to country, based on the location or origin of corresponding author. In all the publications, the study samples consisted of people living in continental Africa. Figure 3A displays the distribution of scientific production in the top five countries. Depicted by the blue line, South Africa exhibits the highest number of publications, which appears to be exponentially increasing at a far more rapid rate compared to others.

Wang et al. (2023) made similar observations in their bibliometric analysis of PP worldwide, with South Africa emerging as a prominent African country, ranked eighth globally in terms of publications. Notably, the other leading countries were the United Kingdom and United States of America, rather than additional African countries. It is important to note that these results reflect countries where a significant amount of research has been conducted but do not imply an absence of PP studies in other African countries. Although we limited our search criteria to African countries, there were instances where a study conducted in Africa includes a corresponding author affiliated to institutions outside Africa. We still included these studies because their samples comprised participants in Africa. Given that the analysis simply shows the affiliation of the corresponding author, it is possible that some of these authors are African researchers who are currently affiliated with Western institutions. Figure 3B provides an overview of the 10 most productive countries in PP and well-being research in Africa. As expected, South Africa had the highest number of publications (1166), followed by the United States (208), the United Kingdom (62), Ghana (42), Nigeria (39) and Kenya (32). The pattern of publications indicates that a substantial amount of work is being carried out in South Africa, often accompanied by Western interests on well-being research in Africa. It appears that the Southern (represented by South Africa) and Western (represented by Ghana and Nigeria) African regions, then followed by some activity in the Eastern region (with Kenya and Uganda) account for most of the productivity in Africa.

Regarding citations by country, the top 10 countries are shown in Figure 4. It was observed that South Africa (3674), United States (862), United Kingdom (228), Netherlands (184), and Germany (166) were the top five most cited countries. In terms of both scientific production and being the most cited country, South Africa held the leading position, indicating that it is the fastest growing country in PP and well-being research on the continent. On the continent, SA is followed by Ghana (135) and Mauritius (58) in terms of citations.

**Figure 5:**
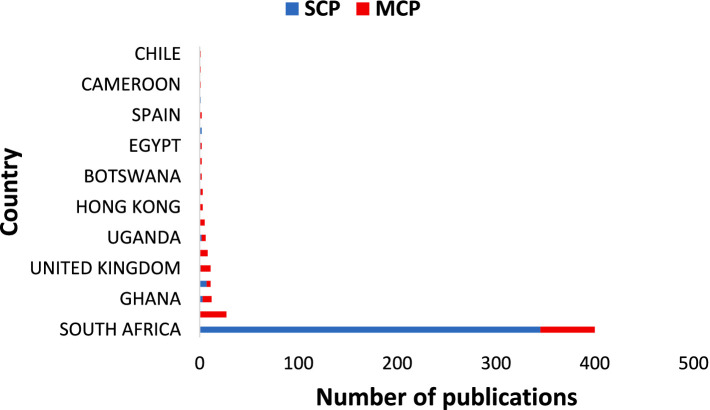
Corresponding author’s countries. SCP and MCP represent intra-country and inter-country collaborations or country publications and multiple-country publications, respectively.

Figure 5 shows the top 20 countries in terms of publications based on the corresponding author. SCP represents the intra-country publication, while MCP represents the inter-county publication. In the case of South Africa, when considering the nationality of the corresponding author, SCP was higher than MCP. For other countries within the top ten, the MCP was much higher, exception of Nigeria.

### Publication by institutions

**Figure 6:**
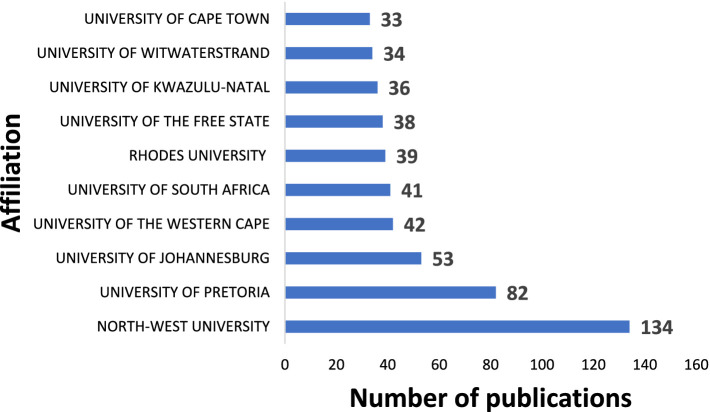
Publications according to institutions.

Figure 6 illustrates the top 10 academic institutions contributing the most to PP and well-being research in Africa. All the ten leading institutions are located in South Africa, with North-West University (134 documents) with the highest number of publications, followed by University of Pretoria (82 publications), and University of Johannesburg (53 publications). The University of Ghana was among the top 20 with 17 publications.

### Publications by journal

**Figure 7:**
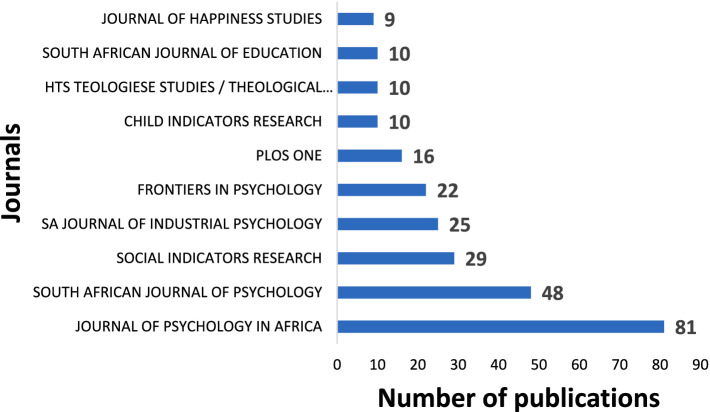
Publications overtime by journal sources.

The top 10 relevant sources (journals) publishing articles on positive psychology and well-being are presented in Figure 7. Generally, it was observed that these top 10 journals published over 200 documents, which accounted for approximately 42% of the total of 622 papers in the sample. According to Figure 7, the Journal of Psychology in Africa (JPA) rank first on the list with 81 documents, making up about 40.5% of the papers published by the top 10 journals. It was followed by South African Journal of Psychology (SAJP) with 48 published documents and Social Indicators Research with 29 documents. The high number of publications in the Journal of Psychology in Africa can be attributed to the journal’s broad scope and a developmental approach to publication success, thus providing an accessible platform for publishing studies from the African continent. Many institutions in South Africa have some agreement with JPA (Taylor & Francis) allowing them to publish at subsidized rates. Given that there is no specialized local journal on well-being, this Journal becomes a good alternative. Furthermore, when examining the top 10 journals, there is a predominance of South African journals with a few international options such as the Journal of Happiness of Studies and Social Indicators Research. Among the top 10 journals, the Journal of Happiness Studies had the lowest number of published documents (*n* = 9) at the time the search was conducted.

**Figure 8:**
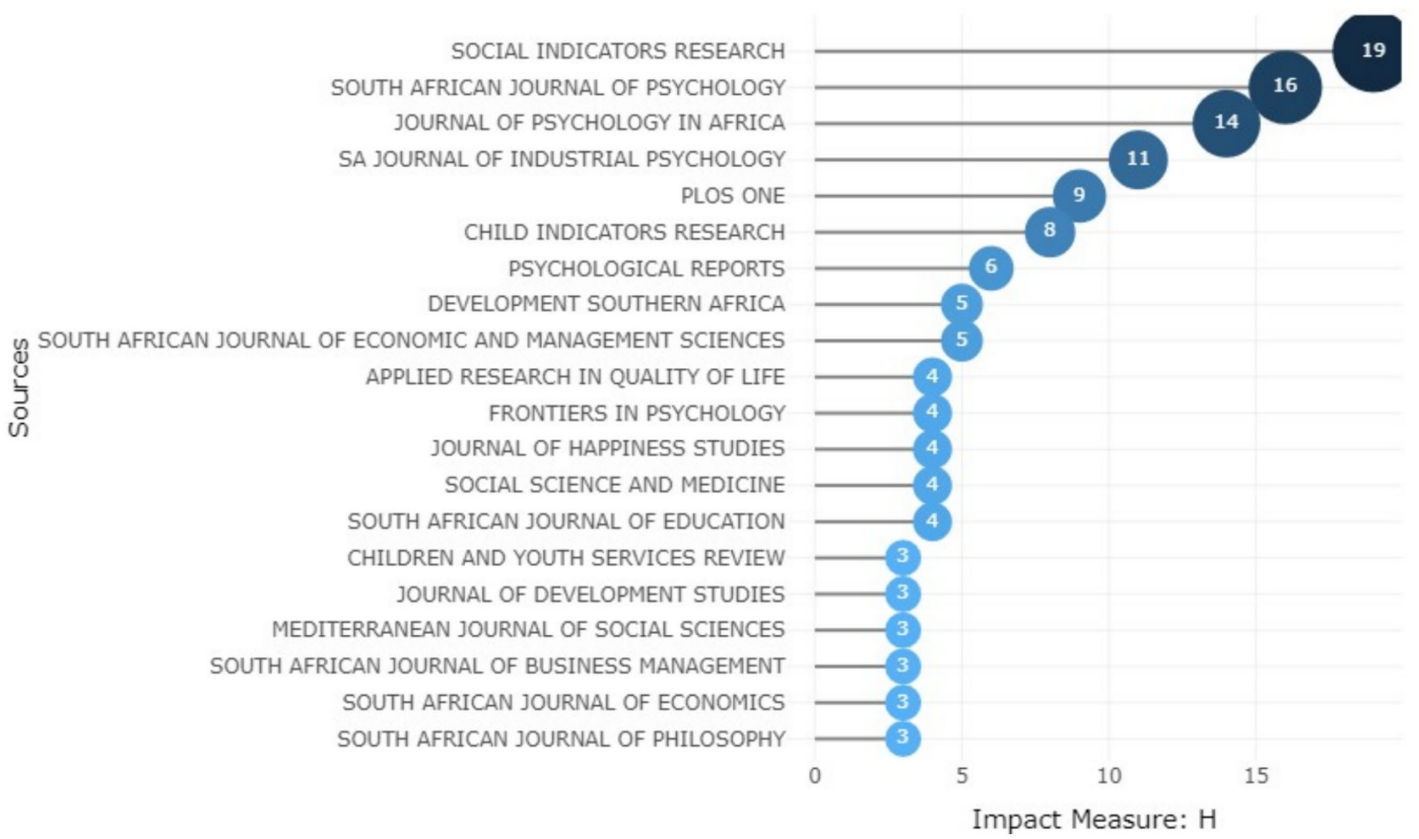
The source impact of scientific publications on positive psychology and well-being.

Figure 8 shows the impact of the relevant sources measured by the h-index. Social Indicators Research (h-index of 19) and South African Journal of Psychology (h-index of 16) top the list, followed closely by Journal of Psychology in Africa (h-index of 14).

It is worth noting that PLoS One and Psychological Reports, which did not appear among the top sources, emerged among the top ten in terms of impact. Conversely, we observed that the Journal of Happiness Studies and Frontiers in Psychology dropped in rank in terms of their impact.

### Most productive authors

**Figure 9:**
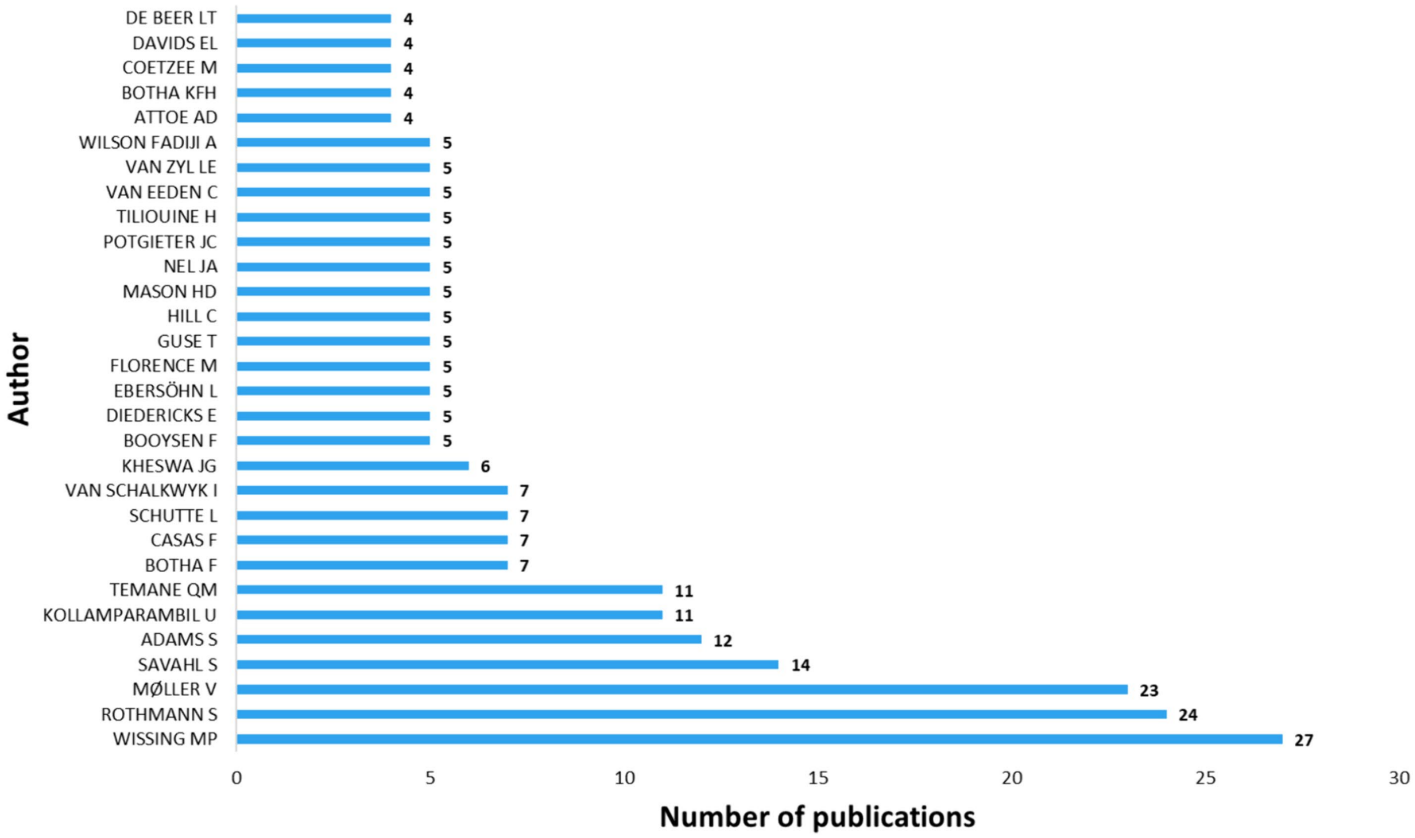
Most productive authors.

From 1983 to 2023, 1,683 authors participated in PP and (psychological) well-being research, with only 136 authors of single-authored documents, constituting 8%. The top 10 most prolific authors are shown in Figure 9. It is notable that Wissing M., Rothmann, S, and Moller V. are the most productive authors, with 27, 24 and 23 relevant publications. Reflecting on the works of these authors, Wissing (2022), for instance argued that the third wave of PP is suitable for understanding the complex nature of bio-psycho-social-ecological well-being and for promoting health and wellness during times of enormous challenges and changes. The paper proposes that future research should increase awareness of metatheoretical assumptions, develop more comprehensive theories to bridge the conceptual fragmentation in the field, and implement methodological reforms while considering context and the interconnectedness of various levels in scientific texts. Rothmann’s work has primarily focused on well-being in the workplace, with his most recent publication addressing predictors of citizenship behaviour and intention to leave (Hennicks et al., 2022). Moller’s research is centered around quality of life and subjective well-being, which we argue is a core component of well-being research in Africa. One of Moller’s most cited articles explores how subjective well-being evolves with age (López Ulloa et al., 2013), supporting the hypothesis of U-shaped relationship between age and subjective well-being. Other notable authors are Savahl, S, and Adams S, each with 14 and 12 publications, respectively. Additionally, Schutte L and Van Schalkwyk I are among the top 10 authors. It is important to note that other highly published researchers in the field, including Guse T, Ebersohn, L, Khumalo I, Mason, H, and Van Eeden, C, may not have emerged in the top rankings due to some of their publications being co-authored with the top 10 authors.

**Figure 10:**
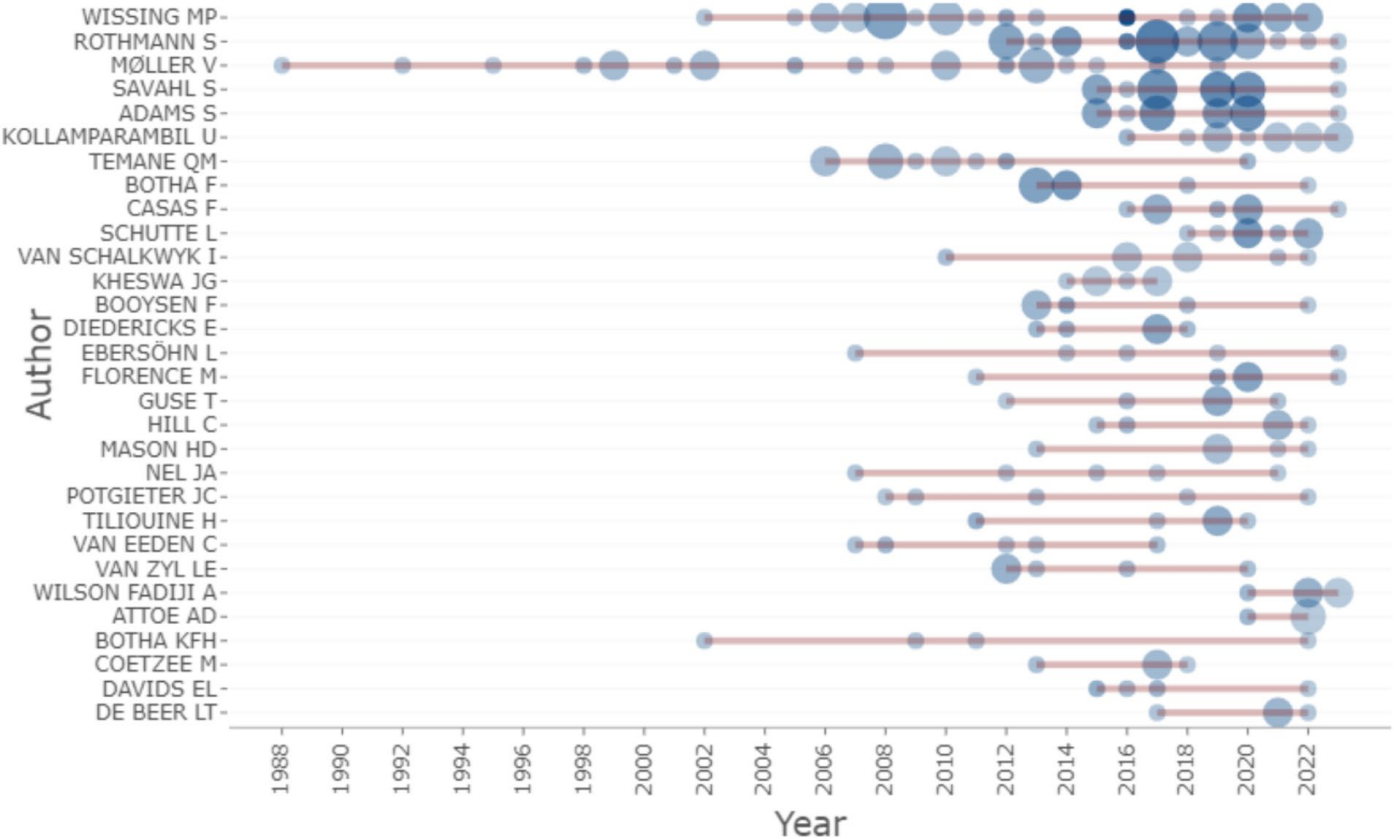
Author’s productivity over time.

Figure 10 shows a graph of the top 10 author’s productivity and impact over time. This graph measures an authors’ relevance over the last 34 years (1983–2023) based on productivity and impact in the subject area. Productivity is determined by the number of articles published by an author in a given period, while impact was assessed based on the number of citations received each year.

In Figure 10, the intensity of the colour is proportional to the year of the citation, and the size of the bubbles represents the annual output of various authors. For instance, Wissing M had approximately 11 total citations per year in 2012 (from 1 document published) and published 1, 3, and 4 articles in 2016, 2017, and 2019, respectively. Rothmann S emerged as the author with the highest production over time followed by Moller, V, Savahl S, and Adam S, with impact scores of 13, 12, 9 and 8, respectively. Between 1983 and 2002 (Figure 10), we see that Moller was the sole source, but from 2002 onwards there is steady growth in research with Wissing M leading. Then Rothmann S follows in 2012. It is reasonable to observe that the study of well-being and PP began to grow significantly about 20 years ago.

Table 1 shows the 10 authors with the most relevant impacts, ordered by h-index. Three measures are provided for the local dataset and the top 10 most productive authors: times cited (TC), h-index (h-index), g-index (g-index), and m-index (m-index; Aithal, 2017). The h-index measures the combination of quantity and quality by comparing an author’s publications to their citations. It serves as a metric for assessing the overall impact of an author’s scholarly output and performance. The g-index is calculated based on the distribution of citations received by an author’s publications, giving more weight to highly cited articles. The last impact measure, the m-index, is another variant of the h-index that represents the h-index per year since an author’s first publication. The m-index is calculated as the h-index divided by the number of years a scientist has been active (Forliano et al., 2021).

**Table 1:** Most relevant author by impact.

Element	h-index	g-index	m-index	TC	NP	PY-start
ROTHMANN S	13	19	1.083	402	24	2012
MØLLER V	12	21	0.333	471	23	1988
WISSING MP	11	21	0.5	491	27	2002
SAVAHL S	9	14	1	197	14	2015
ADAMS S	8	12	0.889	177	12	2015
TEMANE QM	7	11	0.389	174	11	2006
BOTHA F	6	7	0.545	147	7	2013
CASAS F	5	7	0.625	76	7	2016
DIEDERICKS E	5	5	0.455	108	5	2013
SCHUTTE L	5	7	0.833	53	7	2018
VAN EEDEN C	5	5	0.294	86	5	2007
VAN ZYL LE	5	5	0.417	71	5	2012

The most cited authors in the dataset were Wissing M (491 citations), Moller V (471 citations) followed by Rothmann (402 citations). Wissing M, who had the highest author productivity over time (Figure 10), has an h-index of 11, a g-index of 21, and an m-index of 0.5, respectively. On the other hand, Rothmann S, whose publication record began in 2012, has the highest h-index, g-index, and m-index with values of 13, 19, and 1.03, respectively.

### 20 Top most cited document

The top 20 most cited well-being and PP research analysed based on Scopus data is shown in Table 2. These documents have received total citations ranging from 43 to 483. With total citations exceeding 100, Keyes et al. (2008), Ammar et al. (2020), and Delle Fave et al. (2016), were cited the most, receiving 483, 166, and 148 citations, respectively. Keyes et al.’s (2008) work revealed that the Mental Health Continuum–Short Form (MHC–SF) replicated the three-factor structure of emotional, psychological and social well-being in a South African sample. Ammar et al. (2020) examined the impact of COVID-19-related home confinement on well-being. The study revealed that COVID-19 home confinement had a negative effect on mental-wellbeing. Specifically, a significant decrease in the total score of mental well-being was noted. In their study of lay definitions of happiness, Delle Fave et al. (2016) found that definitions of happiness were linked to a broad range of life domains, encompassing both the contextual-social sphere and the psychological sphere. These definitions remained consistent across countries and showed little variation by age and gender. Inner harmony was a dominant theme in psychological definitions, while family and social relationships were central in contextual definitions.

**Table 2:** Top 20 most cited studies.

Authors	Year	Title	Journal	Total citations
Keyes, C. L. M., Wissing, M., Potgieter, J. P., Temane, M., Kruger, A., and van Rooy, S.	2008	Evaluation of the Mental Health Continuum-Short Form (MHC-SF) in Setswana-speaking South Africans	Clinical Psychology & Psychotherapy	513
Ammar, A., Mueller, P., Trabelsi, K. Chtourou, H., Boukhris, O., Masmoudi, L., …. Hoekelmann, A.	2020	Psychological consequences of COVID-19 home confinement: The ECLB-COVID19 multicenter study	PLoS One	206
Delle Fave, A., Brdar, I., Wissing, M. P., Araujo, U., Solano, A. C., Freire, T., Hernández-Pozo, M. D. R., Jose, P., Martos, T., Nafstad, H. E., Nakamura, J., Singh, K., and Soosai-Nathan, L.	2016	Lay definitions of happiness across nations: The primacy of inner harmony and relational connectedness	Frontiers in Psychology	185
Bar-On, R.	2010	Emotional intelligence: An integral part of positive psychology	South African Journal of Psychology	89
van Woerkom, M., Oerlemans, W., and Bakker, A. B.	2016	Strengths use and work engagement: A weekly diary study	European Journal of Work and Organizational Psychology	106
Pieterse, E.	2010	Cityness and African urban development	Urban Forum	67
Posel, D. R., and Casale, D. M.	2011	Relative standing and subjective well-being in South Africa: The role of perceptions, expectations and Income Mobility	Social Indicators Research	64
Schatz, E., Gómez-Olivé, X., Ralston, M., Menken, J., and Tollman, S.	2012	The impact of pensions on health and wellbeing in rural South Africa: Does gender matter?	Social Science & Medicine	76
Westaway, M. S.	2006	A longitudinal investigation of satisfaction with personal and environmental quality of life in an informal South African housing settlement, Doornkop, Soweto	Habitat International	68
Møller, V.	2005	Resilient or resigned? Criminal victimisation and quality of life in South Africa	Social Indicators Research	63
Field, L. K., and Buitendach, J. H.	2011	Happiness, work engagement and organisational commitment of support staff at a tertiary education institution in South Africa	SA Journal of Industrial Psychology	143
Cramm, J. M., Møller V., and Nieboer, A. P.	2011	Individual- and neighbourhood-level indicators of subjective well-being in a small and poor Eastern Cape township: The effect of health, social capital, marital status, and income	Social Indicators Research	52
Møller, V.	1998	Quality of life in South Africa: Post-apartheid trends	Social Indicators Research	49
Khumalo, I.P., Temane, Q.M. and Wissing, M.P.	2012	Socio-demographic variables, general psychological well-being and the mental health continuum in an African context	Social Indicators Research	51
Cheng, C., Jose, P. E., Sheldon, K. M., Singelis, T. M., Cheung, M. W. L., Tiliouine, H., Alao, A. A., Chio, J. H. M., Lui, J. Y. M., Chun, W. Y., Golec de Zavala, A., Hakuzimana, A., Hertel, J., Liu, J.-T., Onyewadume, M., and Sims, C.	2011	Sociocultural differences in self-construal and subjective well-being: A test of four cultural models	Journal of Cross-Cultural Psychology	44
Botha, F.	2014	Life satisfaction and education in South Africa: Investigating the role of attainment and the likelihood of education as a positional good	Social Indicators Research	26
Botha, F., and Booysen, F.	2014	Family functioning and life satisfaction and happiness in South African households	Social Indicators Research	44
de Bruin, G. P., and du Plessis, G. A.	2015	Bifactor analysis of the Mental Health Continuum-Short Form (MHC-SF)	Psychological Reports	46
Rao, N., Singh, C., Solomon, D., Camfield, L., Sidiki, R., Angula, M., … Lawson, E. T.	2020	Managing risk, changing aspirations and household dynamics: Implications for wellbeing and adaptation in semi-arid Africa and India.	World Development, 125, 104,667.	45
Schlebusch, L., Dada, S., and Samuels, A. E.	2017	Family quality of life of South African families raising children with autism spectrum disorder.	Journal of Autism and Developmental Disorders	44
Edwards, S.	2006	Physical exercise and psychological well-being	South African Journal of psychology	44

Outside of the top three cited papers, three more are worth noting for their significant relevance. Posel and Casale’s (2011) work is particularly relevant and highly cited (69 citations). They investigated the relationship between relative standing in terms of income and subjective well-being among South African households. It has been observed that future upward mobility has a smaller effect than upward mobility compared to one’s past, suggesting that life satisfaction is more strongly associated with one’s past accomplishments than with anticipated future success. Other notable works, including Khumalo et al. (2012) (51 citations) focused on translating, validating, and contextualising mental health and well-being instruments as well as examining the extent to which sociodemographic variables predict well-being. Moreover, there has been research addressing quality of life and how different socio-economic factors including safety, crime, living standards impact subjective well-being and quality of life. As shown through researcher progress over time, quality of life and subjective well-being across social indicators research has been spearheaded by Møller, V. This observation is apparent from Table 2 (i.e., Møller, 1998, 2005; Cramm et al., 2011).

In summary, Table 2 displays the most cited studies, which cover a broad range of topics, including the validation of well-being instruments, the impacts of COVID-19 on well-being, the conceptualisation of well-being, emotional intelligence, character strengths, socio-demographic factors and their relationships with subjective well-being and quality of life, as well as research on well-being at the workplace.

## Co-occurrence network analysis

### Co-occurrence—all key words

**Figure 11:**
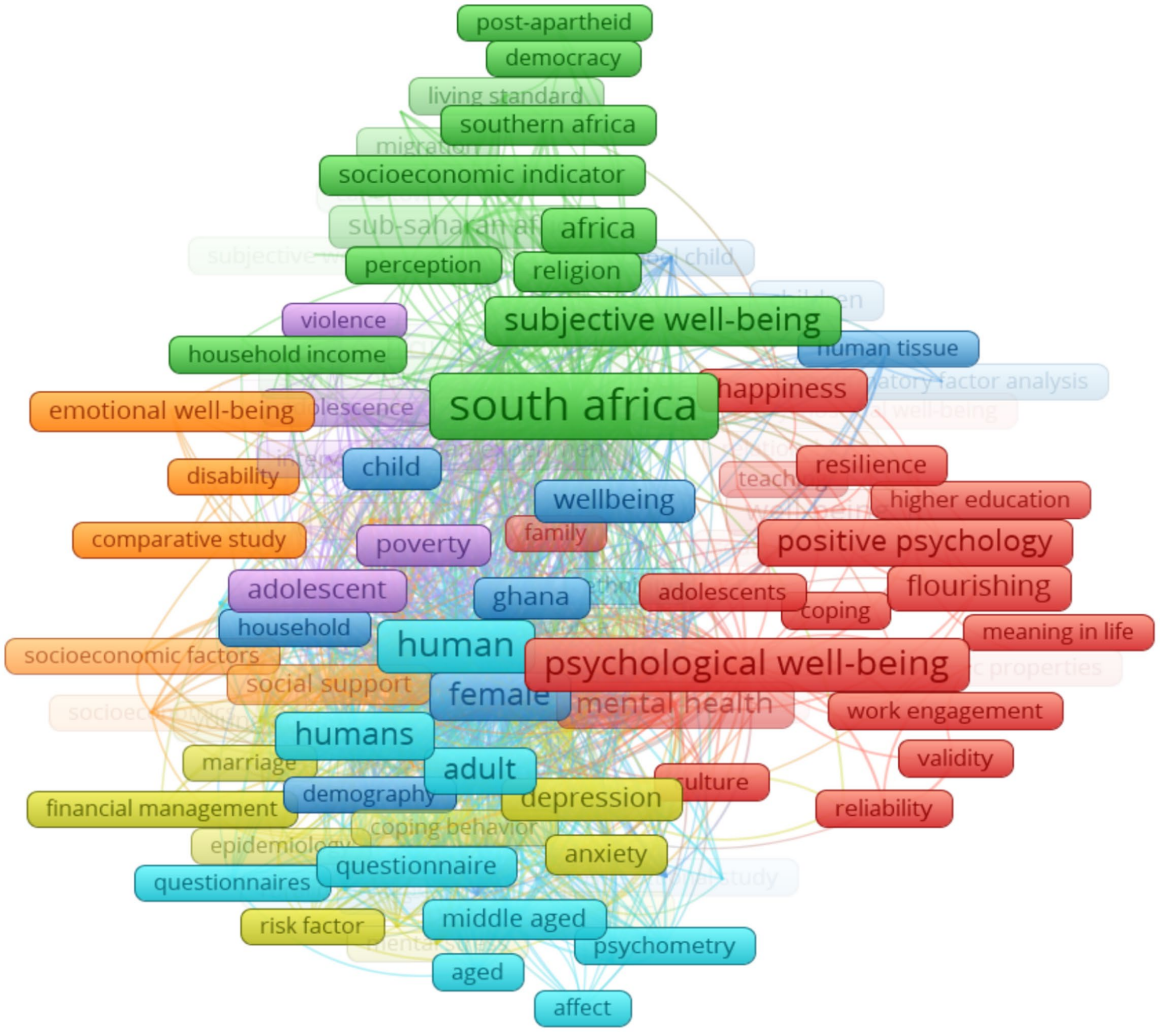
Co-occurrence—all keywords.

The keyword co-occurrence network analysis helps researchers detect the core content of the literature and depict its knowledge structure. It is used to identify “keywords” that co-occur in at least two publications in a period (Zhong et al., 2021). This scientometric method aids in generating clusters that provide a broader view of different research foci within a specific knowledge domain (Rejeb et al., 2021). Keywords are significant part of scholarly publications, playing a vital role in information retrieval and research. In this study, using VosViewer to analyse all keywords (using the full counting method), we selected the minimum number of keywords occurrence as 5, resulting in 146 meeting the thresholds out of the 2,605 keywords. Figure 11 shows the co-occurrence network of all keywords in positive psychology and well-being research in Africa, while the occurrences and link strengths of all keywords are shown in Table 3. Total link strength (TLS) represents the collaboration intensity of keywords. The link strength between the nodes reflects the frequency of co-occurrence of the keywords.

**Table 3:** Occurrences and total link strength of all keywords.

Keyword	Occurrences	Total link strength
South Africa	166	868
Psychological well-being	74	391
Human	69	907
Subjective well-being	58	250
Life satisfaction	52	332
Article	51	733
Quality of life	50	331
Female	48	749
Male	46	715
Humans	46	674
Adult	38	576
Positive psychology	38	70
Well-being	37	89
Mental health	36	305
Africa	32	151
Flourishing	31	70
Controlled study	28	459
Psychology	26	347
Depression	25	259

The total link strength is the sum of the link strengths of the keyword with respect to all the other keywords (Guo et al., 2019; Martynov et al., 2022). As shown in Figure 11, we identified seven clusters of words with distinct colours. The nodes in the figure represent keywords, and the node size corresponds to the co-occurrence frequency of the keyword or the number of publications with the corresponding keywords. The distance between two keywords in the visualization is determined by density, and the higher this density, the closer the distance between the nodes (Rejeb et al., 2021). The red cluster (cluster 1, with 39 items) features ‘psychological well-being’ as the most occurring keyword. In this cluster, we observe a focus on key positive psychological constructs with a good mix of hedonic and eudaimonic well-being perspectives. Notable keywords include: ‘meaning’, ‘hope’, ‘happiness’, ‘flourishing’. There were other key elements in this cluster, such as ‘adolescents’, ‘African context’, and ‘South Africa’, which seem to refer to the contexts or samples in which the PP constructs were studied. Cluster 2, represented by green, contained 24 items, with ‘South Africa’ as the most frequently occurring keyword. In this cluster, there is a mixture of PP constructs, including ‘subjective well-being’, ‘quality of life’, and ‘life satisfaction’, as well as psychosocial factors, such as democracy, income, living standards, migration, and other, which potentially serve as antecedents of subjective aspects of well-being. Cluster 3 has female as the most frequently occurring keyword, with total items of 20 in this cluster. This cluster shows examples of demographic characteristics that are commonly studied in PP and well-being research. It also includes information on various research designs, including randomized controlled trial, cross-sectional studies, and confirmatory factor analysis. In cluster 4 (yellow), which consists of 18 items, the focus appears to be on examples of indicators of psychological distress such as stress, anxiety, depression, mental stress etc.

Cluster 5 is represented by the color purple, with ‘adolescent’ as the most commonly occurring keyword. This cluster comprises 17 items, and its central theme revolves around ‘physical health’. Other keywords within this cluster include health status, HIV/AIDS, poverty, crime and violence. Clusters 6 and 7, represented by light blue and orange, consist of 16 and 12 items, respectively. ‘Human’ is the most frequently occurring word in cluster 6, while ‘article’ is the dominant keyword in cluster 7.

Table 3 shows the top 10 keywords in the all-keyword co-occurrence analysis. It is evident that the keyword with the highest occurring keywords in clusters 1 to 3 also appear in the top 10. ‘South Africa’ had the highest frequency (166), followed by ‘psychological well-being’ (74), ‘human’ (69), and ‘subjective well-being’ (58).

### Co-occurrence—author keywords

**Figure 12:**
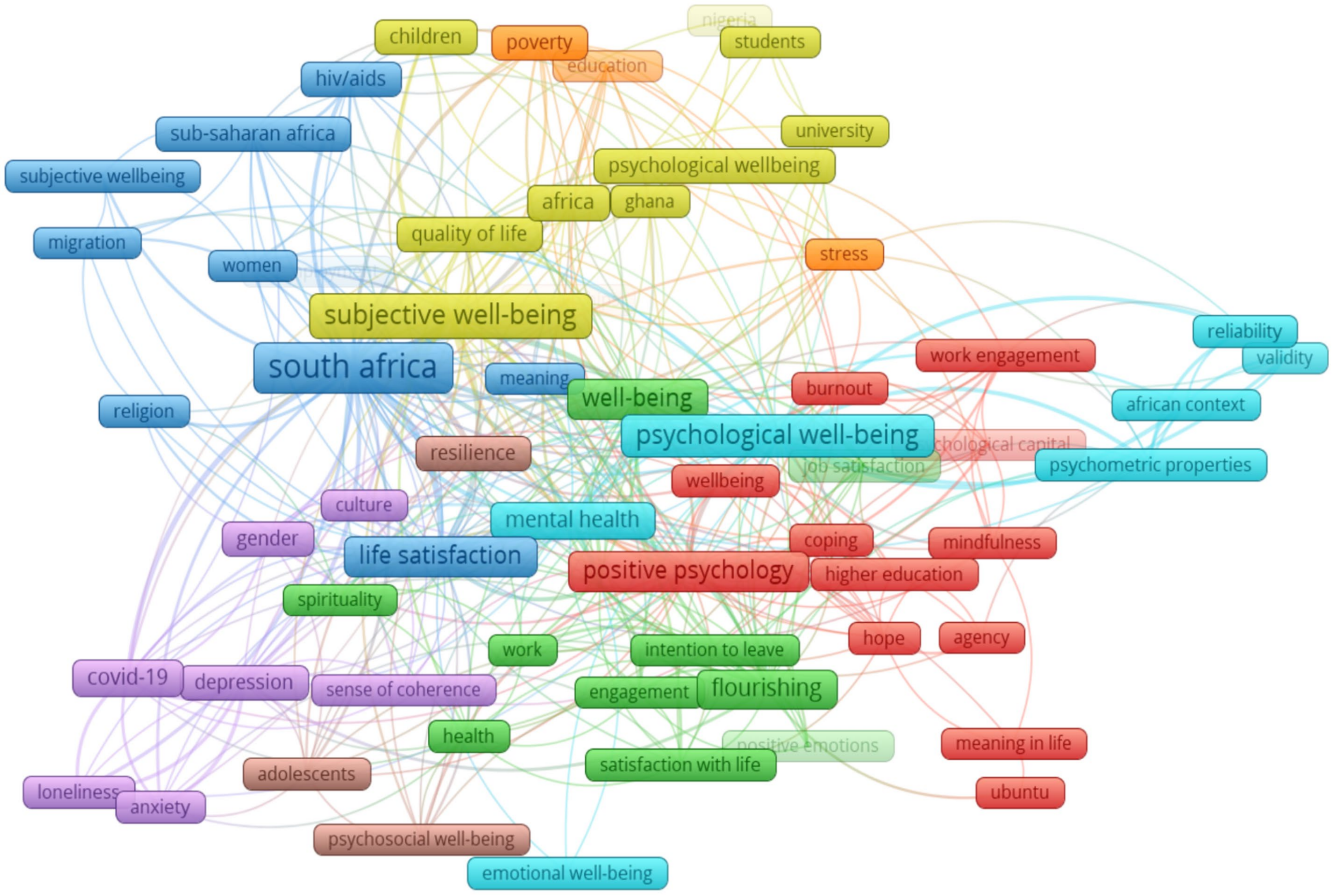
Co-occurrence—author keywords.

Author keywords are those provided by the original authors. For this analysis, we selected the minimum number of keyword occurrences as 5, resulting in 63 keywords that met the thresholds out of 1792. Figure 12 shows the co-occurrence network of author keywords in positive psychology and well-being research in Africa. This network consists of 8 clusters, each represented by distinct colours. Notably, the red cluster (cluster 1, 12 items) includes keywords such as positive psychology, well-being, mindfulness, hope, agency, meaning in life, work engagement, and more, all of which are closely related to the topic of “well-being and positive psychology in Africa.” Within this cluster, positive psychology is the frequently occurring keyword. The green colour represents cluster 2 (11 items) with well-being as the most frequently occurring keyword. This cluster also includes keywords like engagement, flourishing, happiness, satisfaction with life, spirituality etc. Cluster 3 is represented by the colour blue and contains 10 items with keywords such as subjective well-being, quality of life. Africa, Ghana, psychological well-being, students, and others. The most common keyword in this cluster is ‘South Africa’. In cluster 4 (yellow colour; 9 items), ‘subjective well-being’ stands out as the highest occurring keyword, accompanied by keywords such as Africa, Ghana, quality of life, students, and more. In cluster 5, (purple; 7 items), COVID-19 presented as the most frequently occurrence keyword, along with loneliness, anxiety, depression, sense of coherence, etc. Clusters 6, 7, and 8 represented by light blue, orange, and light brown colours, respectively, each with 7, 4, and 3 items, respectively. In cluster 6, keywords such as emotional well-being, reliability, validity, and psychometric properties were prominent. Cluster 7, on the other hand, comprised keywords such as education, poverty, relationships, and stress. Of note, resilience was unique to cluster 8.

Table 4 shows the top 10 keywords from the co-occurrence author-keyword analysis. The most frequently occurring keywords included positive psychology, psychological well-being, South Africa, and subjective well-being. Similar to the all-keywords analysis, South Africa (112), subjective well-being (57), psychological well-being (54) and positive psychology (38) were the most frequently occurring terms and the highest link strength. Consistent with other findings, the pattern of author keyword occurrences shows that more research has been conducted in South Africa. Table 4 also shows that ‘poverty’ and ‘HIV’ had the lowest occurrences and total link strength. The total link strength indicates the number of publications in which two keywords occur together, implying that these keywords are not central to PP research in Africa.

**Table 4:** Occurrences and total link strength of author keywords.

Keyword	Occurrences	Total link strength
South Africa	112	161
Subjective well-being	57	81
Psychological well-being	54	80
Positive psychology	38	53
Well-being	37	58
Life satisfaction	36	60
Flourishing	31	48
Mental health	22	45
Happiness	20	36
Covid-19	20	34
Africa	15	9
Depression	14	34
Quality of life	14	29
Resilience	13	25
Gender	12	17
Children	12	13
Sub-Saharan Africa	11	16
Poverty	10	22
HIV/AIDS	10	21

### Co-occurrence—index keywords

**Figure 13:**
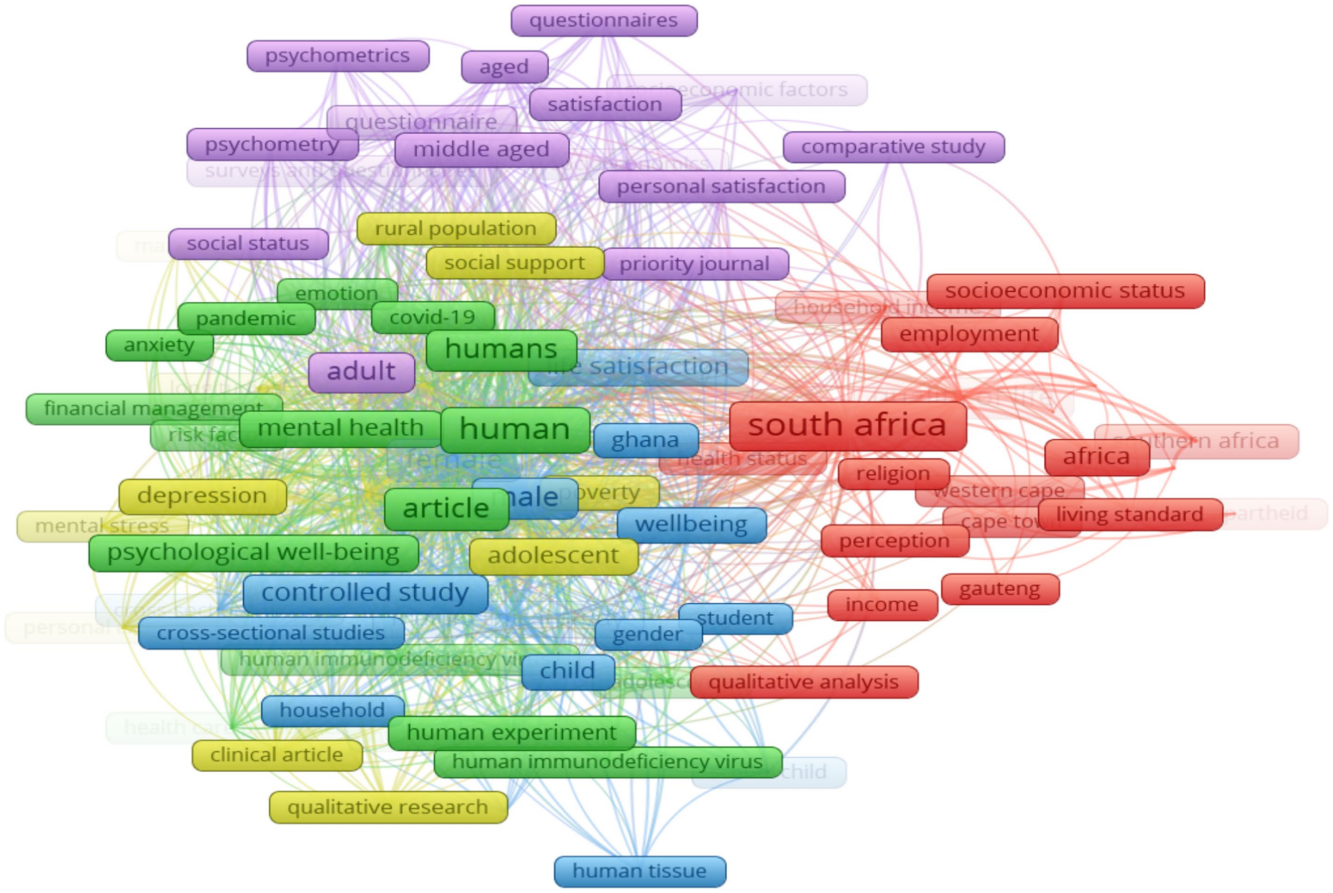
Co-occurrence—index keywords.

Scopus selects index keywords standardized to vocabularies derived from thesauri owned or licensed by Elsevier. Unlike author keywords, index keywords consider synonyms, alternate spellings, and plurals (Golub et al., 2020). For the index keywords, we selected a minimum occurrence as 5, of which 88 met the thresholds out of the 1,001 keywords. The co-occurrence network of index keywords in well-being and positive psychology research is shown in Figure 13. The co-occurrence network contains five clusters. Cluster 1, represented in red, contains 22 items with keywords such as South Africa, employment, religion, living standards, health status and qualitative data analysis. In this cluster, South Africa was the highest occurring keyword and part of the top 10 index keywords occurrences (Table 5). The green cluster represents the second cluster (18 items) with humans as the highest occurring keyword. Other keywords in this cluster include psychological well-being, mental health, emotions, COVID, anxiety, financial management, HIV etc. Cluster 3, represented in blue, comprises 16 items with ‘female’ as the keyword with the highest occurrence. Other keywords in cluster 3 include controlled study, gender, cross-sectional studies, randomised controlled trials, etc. The yellow cluster (Cluster 4), consisting of 16 items, containing keywords such as depression, coping behaviour, mental stress, poverty etc. In this cluster, the occurrence of psychology was the highest. The last cluster (cluster 5, 16 items), represented by purple has ‘adult’ as the highest occurring keyword.

**Table 5:** Occurrences and total link strength of index keywords.

Keyword	Occurrences	Total link strength
South Africa	91	632
Human	69	809
Article	51	658
Female	48	674
Male	46	643
Humans	46	615
Quality of life	42	254
Adult	38	526
Controlled study	28	410
Psychology	25	309
Adolescent	23	339
Africa	21	90
Psychological well-being	20	272
Mental health	20	236
Major clinical study	19	258
Life satisfaction	19	197
Child	17	226
Wellbeing	15	173
Depression	14	213

Table 5 shows that ‘South Africa’ (91) is the highest occurring index word. This followed by non-field specific words like female, male, human and article. ‘Quality of life’ (46) closely follows as a well-being construct with high occurrence. ‘Psychological well-being’ (21) only appears closer to bottom of the list.

### Thematic map and evolution

**Figure 14:**
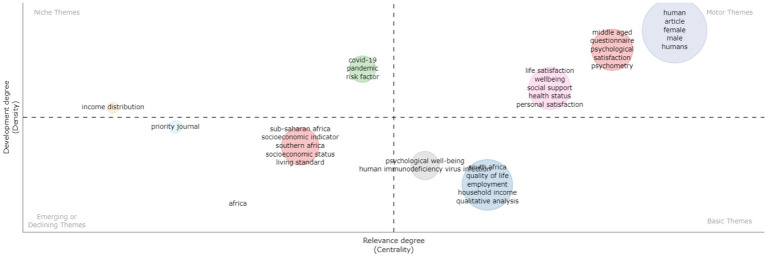
Thematic map and evolution.

Figure 14 displays the thematic map of the keyword and dataset under investigation in this study. The map is divided into four quadrants: Niche themes, Motor themes, Basic themes, and Emerging themes, categorized based on their relevance degree (centrality) and development degree (density). For the analysis, we set the number of words at 1000, the minimum cluster frequency (per 170 thousand documents) at 5, the number of labels at 5, and used the “Walktrap” algorithm for clustering. As seen in Figure 14, the Motor themes quadrant (upper right) demonstrated greater density and relevance. It highlights topics such as well-being, life satisfaction, social support, health status, psychometry, and personal satisfaction. Given that these constructs, concepts, and contexts are at the core of PP research, they continue to merit in-depth examination and further research. The Niche themes quadrant, comprising topics of covid-19 and income distribution, show high development degree but low centrality (i.e., relevance), which suggests that they are not central to well-being research. The Basic theme quadrant (lower right) contains topics such as psychological well-being, quality of life, employment, household income, qualitative analysis and HIV. These topics have relatively high development and relevance degrees. While the trend topics in the upper right quadrant are the most promising for future research, the topics in the lower right quadrant (basic themes) also offer favourable research prospects and are worth further investigation. Furthermore, the Emerging themes quadrant (lower left) contains topics such as the sub-Saharan Africa, socio-economic indicator, living standards and Southern Africa. These emerging topics exhibit low development and relevance degrees because they currently do not seem to be at the core of well-being research in Africa.

## Identification of a research agenda (opportunities and gaps) in well-being and positive psychology

Well-being and positive psychology research, arguably, focus on positive experiences and psychological functioning as indicators of well-being. As we have already highlighted in the introduction, at least three waves of positive psychology have emerged, each building on theoretical, research, and practice gaps (Lomas et al., 2021; Wissing, 2022). Our analysis to some extent reflects the first, second and third waves of PP. Specifically, when we closely examine motor themes, which represent established and relevant research, there is a general focus on the individual, indicative of a first wave characteristic. The lack of mention of socio-cultural factors is noteworthy. However, the basic themes reflect an inclusion and consideration of contextual factors, easily situated in the second wave, and these topics are still in their initial phases of development. Examples clearly include employment, household income, and quality of life. It is also important to interrogate why socio-economic indicators and living standards seem to have little relevance and seemingly declining in the focus of current research. Nonetheless, the emergence of sub-Saharan Africa is an indication of regional integrative and collaborative research efforts beyond the national boundaries. In line with these themes and the considerations in the third wave of positive psychology and well-being research, we highlight some opportunities and gaps for future considerations.

### Clarity on conceptualisation of well-being/psychological well-being

One ongoing criticism of well-being and positive psychology research is the lack of clarity of terminologies and conceptualisations of these constructs (Iasiello et al., 2023; Van Zyl et al., 2023). This issue is evident in the diverse indicators used as proxies of well-being. It subsequently has implications for operationalisation and measurement (Iasiello et al., 2023). In their compilation of positive psychology research in Africa, Wilson Fadiji and Wissing (2022) noted that it was challenging to clearly delineate positive psychology research. Some authors use the term ‘well-being’, but there was minimal reference to relevant theoretical conceptions of well-being. These studies often employed negative indicators in their well-being measurements and simply added a measure of subjective well-being, such as life satisfaction. This gap is not unique to research in Africa but reflects of a broader research gap in this field.

Related to the lack of conceptual clarity, there is also a shortage of culturally-relevant measures of well-being (Cooke et al., 2016). Researchers have struggled to reach a consensus on the essential components of well-being, resulting in underdeveloped measures to capture the construct. Many existing conceptualisations of well-being are extensional in nature. The use of extensional definitions of positive mental health has resulted in a plurality of partially overlapping multi-dimensional models, which are often circularly defined by other multi-dimensional concepts (Iasiello et al., 2023). Consequently, there is the need for ongoing research to delve into the concept of well-being. This acknowledgment is notwithstanding present innovative developments in contexts such as Ghana where researchers (e.g., Osei-Tutu et al., 2020; Wilson Fadiji et al., 2021; Osei-Tutu et al., 2022) have used mixed methods to explore well-being in response to the contextual landscape.

### Further exploration of contextual factors (beyond income) and country levels of well-being

An examination of both the relevant and developed themes reveals that researchers have explored socio-economic indicators as contextual factors influencing well-being. One of the extensively researched contextual predictors is income (Møller, 2013; Lomas, 2023), which is unsurprising given its relevance to subjective well-being and quality of life in the African context. The literature in Africa also highlights other contextual factors such as education, religion, and marital status (Addai et al., 2013; Sulemana, 2015; Wilson and Somhlaba, 2018). However, most of these studies have focused on these indicators in relation to subjective well-being and not eudaimonic well-being.

We contend that an important gap in the African context lies in the need for a deeper understanding of how various contextual factors predict individual functioning. For instance, does marital status or belonging to specific ethnic groups predict higher psychological functioning? This understanding will facilitate the development of more nuanced and better targeted well-being promotion tailored interventions, which take into consideration the many forms of culture (Cohen, 2009) shaping peoples’ lives aimed at specific groups that require well-being promotion. While happiness levels are undeniably crucial, research has shown a strong link between improved functioning and various aspects, including subjective well-being and physical health. More so, there is a need for research that assesses national levels of flourishing using both subjective well-being and eudaimonic indicators. Except for international surveys (Oishi and Diener, 2014), there is almost no available evidence of national-level flourishing data in other less researched-African countries. Such data could help identify areas of well-being research and interventions.

The present study also highlights the importance of interdisciplinary collaboration and the integration of diverse perspectives in research endeavours. This interdisciplinary approach would be particularly relevant in the context of pandemic (e.g., the COVID-19 pandemic), where the mental health impacts extend beyond individual psychological factors to include social, economic, and environmental determinants of well-being (Xiong et al., 2020). Drawing from the findings of the present study, one approach for preparing and mitigating future pandemic would be to invite experts from various fields such as psychology, public health, sociology, and economics, researchers, and policymakers to collaborate to develop comprehensive and holistic strategies and programmes to promote well-being and build resilience of the African peoples, targeting particularly the general, non-clinical but vulnerable population groups.

### Better “Africa representativeness” and “gathering big data”

We also observed that PP and well-being research on the continent is more concentrated in South Africa. While there are shared cultural experiences, there is a need for broader representation of countries in well-being studies. This is because unique socio-cultural factors may exist that cannot be extrapolated from the Southern African context to other parts of Africa. Appiah (2022a,b) describes several sociocultural, theoretical, and methodological issues that can potentially constrain the design, uptake, and effectiveness of PPIs in the African context. In the chapter by Wilson Fadiji and Wissing (2022), the authors noted that despite shared experiences, countries on the continent still grapple with unique historical issues and cultural practices that have the potential to shape their well-being experiences. Africa also faces a real present-day reality of underdevelopment, conflict, poverty, and brain-drain.

In terms of gathering “big data,” there is the potential for new research. Real-time data on happiness levels has been collected using social media platforms (Marengo et al., 2021). Other studies have analysed people’s feelings and perceptions about specific events using tweets. These platforms provide access to large data sets that capture levels and experiences of well-being in real-time. Furthermore, such research can involve counting of affective words in written documents and sentiment analysis of words in twitter feeds, Facebook posts or deriving opinion polls using common devices like mobile phones and apps. Such big data can also retrieve on an organisational level as well as about government services such as health and transportation. While examples of such work are common in the West, they are not prevalent in the African context. We acknowledge that such platforms may not cover rural communities; however, valuable data can be obtained from those who have access to such technology.

### Research and policy standpoints

The present study identifies thematic clusters and developmental patterns of well-being and positive psychology research in Africa, with potential important research and policy implications. Firstly, the emphasis on contextual factors influencing well-being and the call for a more nuanced understanding of bio-psycho-social-ecological well-being highlight avenues for interdisciplinary collaboration and the integration of diverse perspectives in research endeavours. Secondly, the study acknowledges the limitations of Western theoretical terms in capturing the richness of African experiences, underscoring the importance of cultural sensitivity and inclusivity in research design and terminology. Thirdly, the recognition of South Africa’s dominance in positive psychology research should prompt policymakers to consider strategies for enhancing research capacity and collaboration in other African countries. A potential strategy building research capacity of scholars across the continent would be to encourage intercountry collaborations and knowledge-sharing initiatives to foster a more holistic understanding of well-being and facilitate the development of culturally relevant intervention programmes (Appiah, 2022a,b). Lastly, the finding emphasising on concept clarification and the exploration of social indicators of well-being underscores the need for policy frameworks that address the multifaceted nature of well-being beyond traditional measures. There is urgent need for researchers and policymakers to integrate indicators such as class, region, and religion into research and policy frameworks in order to design more targeted and inclusive interventions that address the unique needs of different population groups.

In addition to the dimensions of well-being discussed in our study, we also acknowledge the contribution of other factors that have shaped our understanding of well-being in the African context. Factors such as spirituality, community cohesion, and resilience have played significant roles in shaping the conceptualisation, experience, and assessment of well-being among African populations. Spirituality, deeply ingrained in many African cultures, often serves as a source of strength, comfort, and meaning, contributing to overall well-being (Selman et al., 2013; Arrey et al., 2016). Similarly, the strong sense of community and collective identity prevalent in African societies fosters social support networks and resilience in the face of adversity (Raniga and Mthembu, 2017). Additionally, the socio-political landscape and historical experiences of colonialism and post-colonialism have profound implications for mental health and well-being in Africa, highlighting the need for contextually sensitive interventions. Other dimensions such as the relationship between environmental sustainability, biodiversity, and human well-being, as well as the relationship between socioeconomic factors and well-being outcomes across different regions and population groups (Imasiku et al., 2020), carry important significance in determining and promoting well-being and health, more generally, in the African context. Yet, well-being remains a complex construct and researchers have continued to contribute to expand our understanding, conception, and its assessment. For instance, Yildirim and Belen’s (2018) study on fear of happiness introduces a novel perspective on well-being by examining the role of psychological factors such as fear in predicting subjective well-being. This finding underscores the importance of considering individual differences and psychological traits in understanding well-being outcomes, as advocated by the present study’s call for a more nuanced approach to well-being research. Again, Yıldırım’s (2019) work on irrational happiness beliefs explores the conceptualisation and measurement of this construct and its relationship with various factors such as personality, coping strategies, and arousal. This study adds to the understanding of subjective well-being by highlighting the role of irrational beliefs in shaping individuals’ perceptions of happiness and overall well-being. By acknowledging the influence of cognitive factors on well-being, researchers and policymakers can develop interventions that target maladaptive thought patterns to enhance subjective well-being.

### Limitations of the study

The current bibliometric review has some limitations that needs to be mentioned. Since the search was conducted using a particular search database, Scopus, only studies available at that time as well as those indexed by Scopus were included in the analysis. This would imply that studies not included in the Scopus database might not be reflected in the review.

In addition, although we made efforts to include as many relevant conceptual words, phrases, and terminologies that related to positive psychology and well-being, it is possible that some key studies may have been inadvertently omitted. An example will be using emotional well-being or positive affect in place of happiness, which might have led to the exclusion of some studies. Nevertheless, it is important to note that our findings reflect current research trends on the continent. For instance, the number of citations and authors’ impact measurements can change frequently, but our findings still identify key authors in the field. This also applies to the number of publications during the period under consideration. Furthermore, the scope of the analysis covered by bibliometrix does not provide information on research methodologies within the region. In addition, although we provide information on the most productive countries, our analysis could not draw out the samples/contexts of research for the authors outside of the Continent.

## Conclusions and recommendations

The present study involved a bibliometric analysis of PP research and related well-being constructs in Africa to better understand the field’s current condition, hotspots of research, and thematic developmental patterns. Our findings demonstrate a gradual but steady growth in well-being research on the continent, although skewed towards South Africa and notable researchers in this space. Our analysis shows that the field is currently in a period of rapid development which is in alignment with international trends and evidenced by its presence in internationally accredited Journals.

We note that current terminologies that dominate PP research also guide work on the continent. However, we also note some studies are unintentionally excluded when purely Western theoretical terms are used. For instance, studies on a ‘life worth living’ or ‘the good life’ tends to be missing out searches that used predominantly Western concepts. Future research can consider a more versatile set of terminologies. Additionally, some notable authors might have failed to appear, an example Theron, L who has done extensive research on resilience. In identifying the gaps in the current state of research, we highlight concept clarification, moving beyond South Africa and more exploration of social indicators of well-being such as class, region, religion. In terms of moving beyond South Africa, it would be interesting to see other hotspots of research if South Africa is excluded from the list of African countries.

## Author contributions

AW: Conceptualization, Formal analysis, Methodology, Software, Writing – original draft. IK: Writing – review & editing. MW: Conceptualization, Writing – review & editing. RA: Writing – review & editing.
